# COV-OBS.x2: 180 years of geomagnetic field evolution from ground-based and satellite observations

**DOI:** 10.1186/s40623-020-01194-2

**Published:** 2020-10-23

**Authors:** Loïc Huder, Nicolas Gillet, Christopher C. Finlay, Magnus D. Hammer, Hervé Tchoungui

**Affiliations:** 1 Univ. Grenoble Alpes, Univ. Savoie Mont Blanc, CNRS, IRD, IFSTTAR, ISTerre, 38000 Grenoble, France; 2 DTU Space, National Space Institute, Technical University of Denmark, Elektrovej 327, 2800 Kgs Lyngby, Denmark

**Keywords:** Geomagnetic field, Secular variation, Stochastic equations, Model uncertainties

## Abstract

We present the geomagnetic field model COV-OBS.x2 that covers the period 1840–2020. It is primarily constrained by observatory series, satellite data, plus older surveys. Over the past two decades, we consider annual differences of 4-monthly means at ground-based stations (since 1996), and virtual observatory series derived from magnetic data of the satellite missions CHAMP (over 2001–2010) and Swarm (since 2013). A priori information is needed to complement the constraints carried by geomagnetic records and solve the ill-posed geomagnetic inverse problem. We use for this purpose temporal cross-covariances associated with auto-regressive stochastic processes of order 2, whose parameters are chosen so as to mimic the temporal power spectral density observed in paleomagnetic and observatory series. We aim this way to obtain as far as possible realistic posterior model uncertainties. These can be used to infer for instance the core dynamics through data assimilation algorithms, or an envelope for short-term magnetic field forecasts. We show that because of the projection onto splines, one needs to inflate the formal model error variances at the most recent epochs, in order to account for unmodeled high frequency core field changes. As a by-product of the core field model, we co-estimate the external magnetospheric dipole evolution on periods longer than 2 years. It is efficiently summarized as the sum of a damped oscillator (of period 10.5 years and decay rate 55 years), plus a short-memory (6 years) damped random walk.
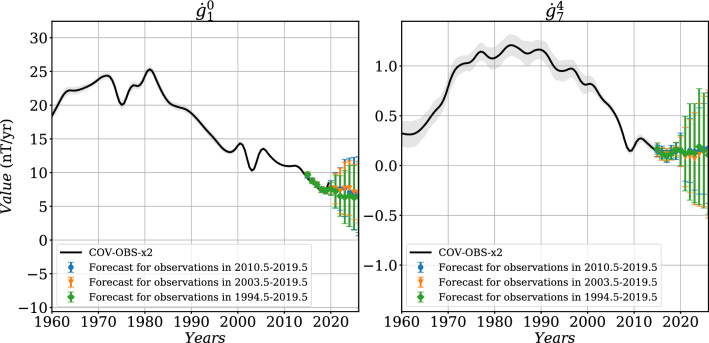

## Introduction

Two crucial characteristic time-scales of the geodynamo are the Alfvén time ($\tau _A\approx$ 4 years based on the propagation of torsional waves, see Gillet et al. 2010) and the turn-over time ($\tau _U\approx 200$ years based on the amplitude of core flow motions, e.g., Finlay et al. 2010). The ratio of these two (the Alfvén number $A=\tau _A/\tau _U\simeq 10^{-2}$) is very difficult to reach with direct numerical simulations of the geodynamo (Schaeffer et al. 2017), due to the wide separation of length and time-scales in the Earth’s outer core. Standard simulations typically reach $A=O(1)$. This motivates the development of parameterizations for small-scale turbulent processes (Nataf and Schaeffer 2015), which makes it possible to numerically simulate the geodynamo at conditions closer to Earth-like (Aubert et al. 2017), reducing the Alfvén number down to $A\approx 0.15$ (Aubert and Finlay 2019).

In this context, there is a need for geomagnetic field models capable of covering both interannual and decadal to centennial changes, while the era of almost continuous satellite records is only two decades long. There is also a need for uncertainty estimates on field model coefficients, if these are to be used as ‘observations’ in geomagnetic data assimilation algorithms (e.g., Fournier et al. 2010; Gillet 2019). The COV-OBS.x2 model presented in this study has been derived in this spirit. It builds upon the earlier COV-OBS models (Gillet et al. 2013, 2015). In practice it is less severely tied to magnetic observations than alternative models covering the recent era such as the comprehensive (Sabaka et al. 2004, 2015) or the CHAOS (Olsen et al. 2014; Finlay et al. 2016) model series. COV-OBS.x2 is a compromise constructed in order to fill a gap between models that focus on the satellite era, and models that cover longer periods such as the historical (Jackson et al. 2000) or archeomagnetic (e.g., Constable and Korte 2015) eras.

We first describe the data used to build the COV-OBS.x2 model and the employed parameterization. We then present how we derive the stochastic a priori information (temporal cross-covariances) used for the model construction, and some distinctions compared with previous generations of COV-OBS models concerning the field induced in the outer core by magnetospheric field changes. Next we show how the COV-OBS.x2 model uncertainties can be used to estimate the probability density function (PDF) of magnetic forecasts within the employed stochastic framework. The predictions for the main field (MF) and its secular variation (SV, the rate of change of the field) result from a best linear unbiased estimate (BLUE) on COV-OBS-x2 Gauss coefficient data.

The obtained model and its associated uncertainties are analyzed, with statistics of the residuals between observation and model predictions, and characteristics in the spectral domain (Lowes spectra and time evolution of Gauss coefficients). We estimate the relative importance of unmodeled core signals at high frequencies in the SV error budget, due to the projection on spline coefficients. We next propose PDFs for 5-year forecasts based on the employed stochastic properties. It is from this method that we derived ISTerre’s candidates models to IGRF-13 (Alken et al. 2020). Finally, we propose a stochastic analysis of external dipole field changes.

## Methods

### Geomagnetic data

We consider below the spherical coordinates $(r,\theta ,\phi )$. Apart from modern satellite data, our data selection process follows closely the one used to construct the COV-OBS.x1 field model. Full details can be found in Gillet et al. (2013, 2015). Here, we only briefly describe the new or updated aspects of the datasets. This only concerns satellite and observatory records over the past two decades or so:

- Satellite data are incorporated by means of virtual observatories (VO) built from the low Earth orbiting CHAMP and Swarm missions. They consist in 4-monthly means, and replace pointwise records from CHAMP and Swarm used in previous COV-OBS models.
- Ground observatory (GO) data are considered through annual differences of 4-monthly revised means after June 1997 (instead of annual means in previous COV-OBS models). We do not consider such revised means at earlier epochs, because some external field corrections are not yet available outside the era of continuous satellite field models.

The choice of 4 months for the binning is motivated by our wish to use, for the inverse problem, an amount of data as much as possible constant through time over the recent era (and 4 months is already significantly less than the time resolution of COV-OBS internal field models). Using higher (e.g., monthly) sampling rates would generate epochs with a smaller number of available VO data, in relation with data selection criteria (see below).

**Fig. 1:**
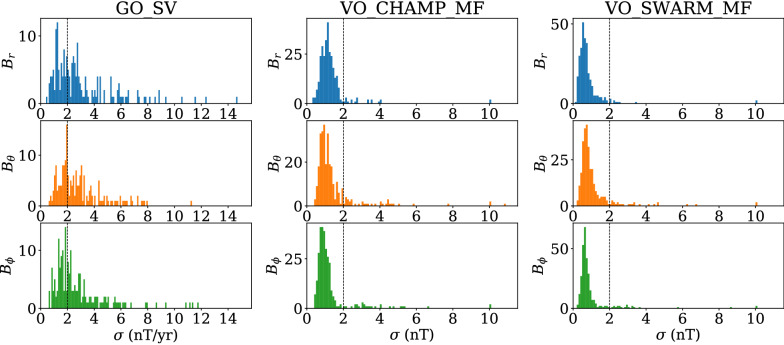
Histogram of observation errors ${\sigma ^{obs}_{GO}}$ and ${\sigma ^{obs}_{VO}}$ for GO and VO datasets, separated by spatial components. The dashed line represents the value of modeling error ${\sigma ^{mod}_{VO}}$ (see text for details). The observation errors for F were derived from these observation errors (see text)

#### Ground observatories data

Up to June 1996 (included), we use the same dataset of ground observatories data as that used in COV-OBS.x1 (annual difference of annual means, with no ionospheric correction). For more recent epochs, we consider instead annual differences of 4-monthly means spanning August 1997 to March 2019. These revised means are computed from hourly mean values provided by the BGS database (Macmillan and Olsen 2013), as described in Olsen et al. (2014). Being constructed upon data that sample all local times, the 4-monthly GO data are corrected for the ionospheric field contribution (and its associated induced counterpart) using the CM4 model (Sabaka et al. 2004). They are not corrected for the magnetospheric contribution as this latter (and its induced counterpart) is co-estimated within the COV-OBS framework throughout the model time-span $[t_s,t_e]=[1840,2020]$ (see below).

To solve the geomagnetic inverse problem, we need to assess uncertainty estimates on 4-monthly GO data. We consider as ‘observation’ errors ($\sigma _{GO}^{obs}$) the uncertainties provided with the three components of GO dataset. For each site, they are estimated as the magnitude of the residuals between GO SV time series and the CHAOS-6 internal plus external predictions (Finlay et al. 2016). Since neither CHAOS-6 nor COV-OBS.x2 parameterize ionospheric sources, we consider here the variance of residuals to GO SV series cleaned for ionospheric contributions. Errors $\sigma _{GO}^{obs}$, shown in Fig. 1, are typically of the order of a few nT/year (ranging from a fraction of nT/year to above 10 nT/year). Note that ‘observation’ errors constructed this way partly account for our current inability to model all magnetic sources. We still inflate these errors by an extra modeling error of variance ${\sigma ^{mod}_{GO}}^2=$ (2 nT/year)$^2$, in order to account for

i. The inability of COV-OBS.x2 to fit SV changes at periods shorter than 2–3 years (see Pick et al. 2019) due to the projection in time onto cubic B-splines with 2 years knot spacing;
ii. The imperfect correction and/or parameterization of external sources. These could lead to biases, especially at high latitudes where slow external changes are difficult to accurately determine. Alternative models could be used as for instance the AMPS model by Laundal et al. (2018), but this is out of the scope of the present work.

Considering that modeling and observation errors are independent, the resulting error budget is then ${\sigma _{GO}}^2 = {\sigma ^{mod}_{GO}}^2+ {\sigma ^{obs}_{GO}}^2$.

#### Virtual observatories satellite data

Instead of the pointwise dataset used for COV-OBS.x1, we use $N_o=300$ VOs derived from the CHAMP and Swarm measurements. VOs consist of processed MF vector data distributed on an equal area grid at the altitude of 370 km for CHAMP and 490 km for Swarm. These are estimated every 4 months from March 2001 to November 2010 for CHAMP, and from November 2013 to July 2019 for Swarm. They are built from selected data (Sun at maximum 10$^\circ$ below the horizon and geomagnetically quiet conditions, see details in Barrois et al. 2018; Hammer 2018). In constructing the VO estimates we use the magnetic data in the $(r,\theta ,\phi )$ frame, rotated from the magnetometer frame using the Euler angles estimates provided by the CHAOS field model (version 6x9), which takes into account star camera attitude errors within its data error budget. Note that each VO datum is derived from hundreds of satellite data within 4 month bins.

These data are cleaned from the lithospheric field contribution, as estimated with the LCS model (Olsen et al. 2017), and from the ionospheric contributions as estimated from the CM4 model (Sabaka et al. 2004). As for GO data, and contrary to what was done in Barrois et al. (2018), they are not corrected for the magnetospheric contribution. Furthermore, in order to reduce the potential impact of field aligned currents, we transform the three-component data ($B_r, B_\theta$ and $B_\phi$) at dipole latitudes higher than $55^\circ$ into intensity data $F=\sqrt{B_r^2+B_\theta ^2+B_\phi ^2}$. As for GO data, we consider two sources of VO data error:

(i) ‘Observation’ uncertainties, of variance ${\sigma ^{obs}_{VO}}^2$. These are estimated for each VO time series separately, based on the variance of the residuals between each series and the predictions of the CHAOS field model after detrending as described in Barrois et al. (2018). In practice $\sigma ^{obs}_{VO}$ are generally less than 2 nT for CHAMP, and slightly less for Swarm, as illustrated in Fig. 1.
(ii) An extra error budget that covers unmodeled error sources, of variance fixed to ${\sigma ^{mod}_{VO}}^2=$ (2 nT)$^2$.

Considering these two error sources as independent, data error variances associated with VO data are thus ${\sigma _{VO}}^2={\sigma ^{obs}_{VO}}^2+{\sigma ^{mod}_{VO}}^2$. At each VO of dipole latitude higher than $55^\circ$, errors on *F* data are deduced from the propagation of errors on ($B_r, B_\theta , B_\phi$) as

1 $$ \begin{aligned} {\sigma _{VO}}_F= & {} \dfrac{|B_r |}{F} {\sigma _{VO}}_r  + \dfrac{|B_{\theta } |}{F} {\sigma _{VO}}_{\theta } + \dfrac{|B_{\phi } |}{F} {\sigma _{VO}}_{\phi }\,.\end{aligned} $$

We finally acknowledge the fact that unmodeled error sources certainly arise from spatially coherent structures, but accounting for spatial cross-covariances in unmodeled external field sources is out of the scope of the present study.

### Parameterization of the COV-OBS.x2 model

The construction of the COV-OBS.x2 field model is largely based on the procedure described in Gillet et al. (2013). We recall here the main common points. In the absence of electrical currents between observation points and the sources (here the Earth’s outer core, of radius $c=3485$ km, and the magnetosphere), the MF derives from a magnetic potential, i.e., $\mathbf{B}=-\nabla (V_i+V_e)$, with $V_i$ and $V_e$, respectively, the internal and external potentials.

The internal potential $V_i$ is expanded on a spherical harmonic basis up to degree $N_i=14$,

2 $$\begin{aligned} V_i(r,\theta ,\phi ,t)= \,&  a\sum _{n=1}^{N_i}\left( \frac{a}{r}\right) ^{n+1} \sum _{m=0}^n\left( g_n^m(t)\cos (m\phi )\nonumber \right. \\&\left. +\,h_n^m(t)\sin (m\phi )\right) P_n^m(\cos \theta ), \end{aligned}$$

with ($g_n^m,h_n^m$) the internal Gauss coefficients of degree *n* and order *m*, $P_n^m$ the Schmidt semi-normalized Legendre polynomials, and $a=6371.2$ km the Earth’s radius. Gauss coefficients are used to define the MF and SV Lowes spectra:

3 $$\begin{aligned} \left\{ \begin{array}{rl} R(n,t)=&{}\displaystyle (n+1)\sum _{m=0}^n g_n^m(t)^2+h_n^m(t)^2\\ S(n,t)=&{}\displaystyle (n+1)\sum _{m=0}^n {\dot{g}}_n^m(t)^2+{\dot{h}}_n^m(t)^2 \end{array} \right. \,. \end{aligned}$$

The external potential $V_e$ accounts for an external axial dipole field in dipole coordinates (plus its induced counter part):

4 $$\begin{aligned} V_e(r,\theta ,\phi ,t)= & {} r\sum _{m=0}^1\left( {\hat{q}}_1^m(t)\cos (m\phi )+{\hat{s}}_1^m(t)\sin (m\phi )\right) P_1^m(\cos \theta )\,. \end{aligned}$$

We use the notations

5 $$\begin{aligned} \left\{ \begin{array}{rl} {\hat{q}}_1^m(t) =&{} \displaystyle q_1^m(t)+\left( \frac{a}{r}\right) ^3g_1^{m\dag }(t)\\ {\hat{s}}_1^m(t) =&{} \displaystyle s_1^m(t)+\left( \frac{a}{r}\right) ^3h_1^{m\dag }(t) \end{array} \right. \,, \end{aligned}$$

with the external ($q_n^m,s_n^m$) and induced ($g_n^{m\dag },h_n^{m\dag }$) Gauss coefficients, and

6 $$\begin{aligned} \begin{bmatrix} {q_{1}^0} \\ {q_{1}^1} \\ {s_{1}^1} \\ \end{bmatrix}(t) = q_{1d}^{0}(t) {\mathbf{m}}(t) \,,\mathrm {with}\; {\mathbf{m}} = \frac{1}{\sqrt{{g_1^0}^2 + {g_1^1}^2 + {h_1^1}^2}} \begin{bmatrix} g_1^0 \\ g_1^1 \\ h_1^1 \\ \end{bmatrix}\,. \end{aligned}$$

A single coefficient, $q_{1d}^{0}(t)$, which describes the external axial dipole in the internal dipole coordinates, is thus used to describe the evolution of the external field. At each iteration *k* of the algorithm (see below), the derivatives involving the forward operator in Eq. (6) are linearized around internal coefficients $g_1^m$ of the previous step $k-1$: we neglect the nonlinearities associated with ${\mathbf{m}}(t)$ when calculating the gradient and Hessian operators (for details, see Gillet et al. 2013). The relation linking ($g_n^{m\dag },h_n^{m\dag }$) to ($q_n^m,s_n^m$) is detailed below.

All internal and external coefficients are expanded in time using order 4 cubic B-splines, with knots every 2 years, spanning the period 1838–2022. A L2 measure of the data misfit is employed, together with a $3\sigma$ data rejection criterion. As a priori information in the inverse problem, we use temporal cross-covariances associated with auto-regressive processes of order 2 (AR-2), as detailed below. Since historical datasets contain some nonlinear data, and because of the relation (6), the model must be sought iteratively. This is done through a Newton–Raphson algorithm, with explicit estimation of the Hessian matrix, starting from the background axial dipole model (see below).

Nevertheless, the parameterization of the COV-OBS.x2 model differs from that of its predecessors:

- We consider an alternative AR-2 type prior for the axial dipole.
- We remove the contribution from the 20 nT background external dipole when estimating the field induced in the core.
- Finally, the prediction over the period 2020–2025, where no data are available, is performed using a BLUE (Best Linear Unbiased Estimate), considering as data sampled realizations of the COV-OBS.x2 Gauss coefficients.

We now further discuss these differences and their motivations.

### Stochastic prior for the axial dipole

In previous COV-OBS models, all internal field Gauss coefficients are considered as realizations of AR-2 processes $\varphi$ governed by a stochastic differential equation of the form (e.g., Yaglom 1962):

7 $$\begin{aligned} d\frac{d\varphi }{dt} + 2\omega d\varphi + \omega ^2\varphi dt = d\zeta (t)\,, \end{aligned}$$

with $\zeta$ a Wiener process. Such two-parameter processes are characterized by auto-covariance functions of the form

8 $$\begin{aligned} C(\tau ) = \sigma ^2\left( 1+\omega |\tau |\right) \exp \left( -\omega |\tau |\right) \,, \end{aligned}$$

with $\sigma ^2=E\left( (\varphi -{\overline{\varphi }})^2\right)$ the variance, ${\overline{\varphi }}=E(\varphi )$ the statistical expectation, and $\omega ^2=E\left( (\partial _t\varphi )^2\right) /\sigma ^2$. We consider that all Gauss coefficients apart from the axial dipole (see below) result from zero-mean AR-2 processes (i.e., their background value is 0).

For the sake of simplicity, we consider a variance of Gauss coefficients, $\sigma _n^2=E\left( {g_n^m}^2\right)$, and a parameter $\omega _n^2=E\left( (\partial _tg_n^m)^2\right) /\sigma _n^2$ that depend only on the degree *n*. We use here this formalism for all coefficients of degrees $n\ge 2$: for these we set parameters $\sigma _n^2$ and $\omega _n^2$ to the same values as in previous COV-OBS models (estimated from the MF and SV Lowes spectra obtained for a satellite field model in 2005, see Gillet et al. 2013). This description was found convenient as it is consistent with the $-4$ slope of the power spectral density obtained for observatory series at periods from 5 to 70 years (De Santis et al. 2003), a feature confirmed later for Gauss coefficient series down to annual periods (Lesur et al. 2017). Indeed, the frequency spectrum of processes defined by Eq. (7),

9 $$\begin{aligned} P(f) = \frac{4\omega ^3\sigma ^2}{\left[ \omega ^2 + (2\pi f)^2\right] ^2}\,, \end{aligned}$$

shows $f^0$ dependence for low frequencies and $f^{-4}$ dependence for frequencies $f\gg \omega /(2\pi )$. This concise description, based on only two parameters per harmonic degree, was validated by the analysis of geodynamo simulations for all coefficients but the axial dipole $g_1^0$ (Bouligand et al. 2016).

Investigations on the frequency spectrum of this latter coefficient instead show a $f^{-2}$ dependence for intermediate frequencies from about $10^{-5}$ to $10^{-2}$ years$^{-1}$. This was observed from both paleomagnetic records (Constable and Johnson 2005; Panovska et al. 2013) and dynamo calculations (Olson et al. 2012; Buffett and Matsui 2015; Bouligand et al. 2016). To account for this effect, we modify the AR-2 prior for the axial dipole $g_1^0$ in COV-OBS.x2, in comparison with previous COV-OBS models.

**Table 1:** Parameters used for the AR-2 processes describing the statistics of axial and equatorial dipoles in the construction of COV-OBS.x2, compared with those used for the COV-ARCH model of Hellio and Gillet (2018), and the earlier editions COV-OBS.x1 (Gillet et al. 2015)

Field model	$\sigma _{g_1^0}$ (nT)	$\sigma _{{\dot{g}}_1^0}$ (nT/year)	$\omega _{g_1^0}^{-1}$ (year)	$\chi _{g_1^0}^{-1}$ (year)	$T_s$ (year)	$T_f$ (year)	$\sigma _{1}$ (nT)	$\sigma _{{\dot{g}}_1^1}$ (nT/year)	$\omega _{1}^{-1} (year)$
COV-ARCH	*6000*	*15*	400	20	*100,000*	63	*3800*	*19*	200
COV-OBS.x2	*7700*	*10*	770	75	*100,000*	235	*4500*	*10*	450
COV-OBS.x1	*17300*	*17*	1014	1014	6370	6370	*17300*	*17*	1014

In italic font the three (resp. two) free parameters for the axial (resp. equatorial) dipole prior. We use the same parameters for $h_1^1$ and $g_1^1$

Following Hellio and Gillet (2018), we consider that the fluctuations of $g_1^0$ (that is ${\tilde{g}}_1^0(t) = g_1^0(t) - {\bar{g}}_1^0$ with ${\bar{g}}_1^0$ the background axial dipole value) are governed by a more general AR-2 process that obeys a three-parameter stochastic equation of the form

10 $$\begin{aligned} d\frac{d\varphi }{dt} + 2\chi d\varphi + {\omega }^2\varphi dt = d\zeta (t)\,, \end{aligned}$$

where $\chi$ and $\omega$ are positive frequencies ($\omega \le \chi$). The auto-covariance function for such a process is

11 $$\begin{aligned} C(\tau ) = \frac{\sigma ^2}{2\xi }\left( (\chi + \xi )e^{-(\chi - \xi )|\tau |} - (\chi - \xi )e^{-(\chi + \xi )|\tau |}\right) \,, \end{aligned}$$

with ${\xi }^2 = {\chi }^2 - {\omega }^2$. The associated frequency spectrum, given by

12 $$\begin{aligned} P(f) = \frac{4\chi \omega ^2\sigma ^2}{\left[ \omega ^2 - (2\pi f)^2\right] ^2 + (4\pi \chi f)^2}\,, \end{aligned}$$

indeed shows a $f^{-2}$ dependence for frequencies in the range (Bouligand et al. 2016)

13 $$\begin{aligned} \left[ f_s, f_f\right] = \frac{1}{2\pi }\left[ \frac{1}{\tau _s}, \frac{1}{\tau _f}\right] = \frac{\omega ^2}{2\pi }\left[ \frac{1}{\chi +\xi },\frac{1}{\chi -\xi }\right] \,. \end{aligned}$$

The $f^0$ and $f^{-4}$ dependencies at, respectively, low ($f \ll f_s$) and high ($f \gg f_f$) frequencies are still present. In the limit $\omega \ll \chi$, the transition periods between the spectrum ranges showing $f^{-4}$, $f^{-2}$ and $f^{0}$ trends are then (Hellio and Gillet 2018):

14 $$\begin{aligned} \left\{ \begin{array}{rl} T_s = f_s^{-1} = 2\pi \tau _s \simeq &{} 4\pi \chi /\omega ^2\\ T_f = f_f^{-1} = 2\pi \tau _f \simeq &{} \pi /\chi \end{array} \right. \,. \end{aligned}$$

**Fig. 2:**
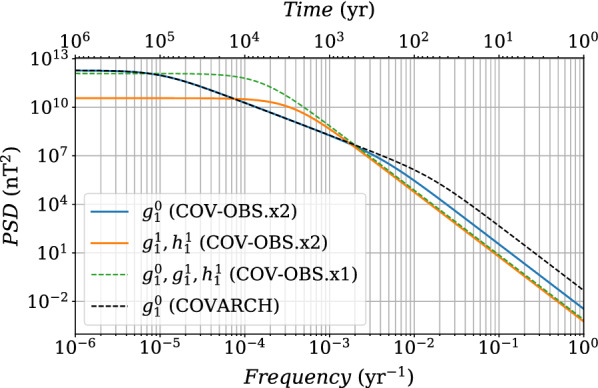
PSD associated with the stochastic processes that define the a priori information used for the construction of COV-OBS.x2 for the axial dipole (blue) and the equatorial dipole (orange) coefficients. Comparison with the corresponding PSD for the axial dipole in COV-ARCH ( Hellio and Gillet 2018, dashed black), and for all dipole coefficients in previous COV-OBS models (Gillet et al. 2013, 2015, dashed green)

Our choice for the values of the three parameters that enter Eq. (11) slightly departs from that made by Hellio and Gillet (2018) for the construction of the archeomagnetic field models COV-ARCH and COV-LAKE (see Table 1). The background value ${\bar{g}}_1^0=-24,000$ nT and the r.m.s. $\sigma _{g_1^0}=7700$ nT are estimated from the average and standard deviation of the axial dipole moment over the past 2 Myr, as estimated with the SINT2000 model (Valet et al. 2005). As described in Additional file 1, alternative estimates are possible (see also Buffett et al. 2013), and our choice of parameters for the axial dipole, relatively conservative, is a compromise between ensuring stability for the axial dipole model and under-estimating rapid dipole changes. We fix $\sigma _{{\dot{g}}_1^0}^2 = E\left( \left( \partial _tg_1^0\right) ^2\right) =$ (10 nT/year)$^2$, which associated with the above choice for $\sigma _{g_1^0}$ comes down to $\omega _{g_1^0}^{-1}=770$ years. As in Hellio and Gillet (2018) we consider $T_s =100$ kyr ($\tau _s=16$ kyr), which fixes the remaining parameter for the axial dipole prior to $\chi _{g_1^0}^{-1}=4\pi /(T_s\omega ^2)\simeq 75$ years, or $T_f\simeq 235$ years. We thus satisfy the condition $\omega \ll \chi$. For the two parameters that define the equatorial dipole statistics, governed by Eq. (7), we choose $\sigma _{1}=4500$ nT, a value consistent with equatorial dipole series in archeomagnetic field models (e.g., Hellio and Gillet 2018, Fig. 6), and $\sigma _{{\dot{g}}_1^1}^2=E\left( \left( \partial _tg_1^1\right) ^2\right) =$ (10 nT/year)$^2$ (with similar values for $h_1^1$), so that $\omega _{1}^{-1} = 450$ years. Our choice $\sigma _{{\dot{g}}_1^1}$ similar to $\sigma _{{\dot{g}}_1^0}$ is in agreement with what is observed in the low viscosity geodynamo simulation by Aubert et al. (2017) or Schaeffer et al. (2017).

In contrast with previous editions of the COV-OBS model, the prior variance and characteristic time for the equatorial dipole now depart from those used for the axial dipole parameters (see Table 1). In this more realistic configuration, the a priori power authorized for axial dipole fluctuations at decadal and shorter periods is larger than that used for COV-OBS.x1, but weaker than that considered for the construction of the COV-ARCH and COV-LAKE models (see Fig. 2). This is the consequence of decreasing $\sigma _{{\dot{g}}_1^0}^2$ (and increasing $\sigma _{g_1^0}^2$), as from Eqs. (12) and (14) one has $P(f)\propto T_s\sigma _{{\dot{g}}_1^0}^4/(\sigma _{g_1^0}^2 f^4)$ towards high frequencies. We discuss further these issues in Additional file 1.

### Parameterization of the induced field

The induced field is anchored to the external field. We consider the core as a perfect conductor, an approximation reasonable since we model only field changes at periods longer than $\approx 2$ years (see Fig. 1 in Olsen et al. 2005). In this framework, the induced field is simply computed by considering that the radial component of the induced field cancels that of the time-dependent inducing field at $r=c$. By differentiating Eq. (4) with respect to *r*, we obtain from Eq. (5)

15 $$\begin{aligned} \begin{bmatrix} {g_{1}^{0\dag }} \\ {g_{1}^{1\dag }} \\ {h_{1}^{1\dag }} \\ \end{bmatrix}(t) = Q_0 \begin{bmatrix} {\tilde{q}}_1^0 \\ {\tilde{q}}_1^1 \\ {\tilde{q}}_1^1 \\ \end{bmatrix}(t)=Q_0 {\tilde{q}}_{1d}^0 {\mathbf{m}}(t)\,, \end{aligned}$$

with $\displaystyle Q_0=\frac{1}{2}\left( \frac{c}{a}\right) ^3\simeq 0.082$. ${\tilde{q}}_{1d}^0 = q_{1d}^{0}(t)-{\overline{q}}_{1d}^{0}$ is the external field perturbation to the background value ${\overline{q}}_{1d}^0=20$ nT. This latter approximately corresponds to the sum of Geocentric Solar Magnetospheric (GSM) and Solar Magnetic (SM) average contributions to the magnetospheric dipole (see Maus and Lühr 2005; Lühr and Maus 2010; Olsen et al. 2014).

The above parameterization slightly differs from that of previous COV-OBS models, where in Eq. (15) ${\tilde{q}}_{1d}^0$ was replaced by $q_{1d}^{0}$ (i.e., the inducing field contained the entire external dipole, even the stationary background). By reducing the core response to only the transient magnetospheric field, we shift in particular the induced axial dipole $g_1^{0\dag }$ by $\approx Q_0{\overline{q}}_{1d}^0\simeq 1.6$ nT (considering a dominant axial with respect to equatorial dipole), and consequently the core dipole $g_1^0$ by the opposite value.

A shift of $\approx 4$ nT was observed between $g_1^0$ in COV-OBS.x1 and most other IGRF candidates (Thébault et al. 2015a, b). The above change to the parameterization of induced fields for the present model COV-OBS.x2 should reduce this shift down to $\approx 2.4$ nT. Our investigations suggest that the remaining difference is most likely associated with the data selection embedded with the satellite data in the COV-OBS framework. In models like CHAOS-6 (Finlay et al. 2016), the SM external field is anchored to indices (such as the Ring Current index RC, see Olsen et al. 2014) that include both calm and disturbed magnetic conditions. The induced field is related to the external field through complex Q-factors (that depend on a mantle conductivity profile). It is thus estimated in the frequency domain before it is transformed back to the time-domain (Maus and Weidelt 2004; Olsen et al. 2005). Constructed as such, it has a zero mean when averaged over all epochs (as it should be if the external signal has stationary properties, but see Velímskỳ and Finlay 2011). Our present external model being computed only from data selected over quiet periods, the above 2.4 nT shift cannot be reduced within the COV-OBS framework.

### Spline-free stochastic forecast of the geomagnetic field

The stochastic 5-year forecast from COV-OBS.x1, candidate model to IGRF-12, was performed by expanding the model time-span (and the support B-spline functions) up to 2020, that is 5 years after the last available data at that time. We see several drawbacks to this procedure. First, there exists a potential for instabilities close to endpoints, associated with the use of splines together with an uneven data coverage (e.g., Gillet et al. 2010). Second, it involves generating a new continuous model for each 5-year prediction. This would imply a rather large computational load when validating our predictions over past periods where the behavior of the field is (to some extent) known. Third, it likely leads to under-estimate the SV error budget, associated with the unmodeled core evolution on short periods, filtered out by the projection onto splines, as discussed in the Results section. We proceed differently, by calculating the BLUE and using

- As data the Gauss coefficients of the COV-OBS.x2 model sampled at a set of epochs,
- As data errors the posterior uncertainties as provided with this model (see Gillet et al. 2013, for the method),
- As prior information, cross-covariances associated with the stochastic processes of each Gauss coefficient.

The prior information in the COV-OBS framework is independent from one coefficient to the other. For the sake of simplicity, we neglect spatial cross-covariances between Gauss coefficient data errors, and predictions are thus operated separately for all Gauss coefficients. In detail, the procedure is the following. For each (*n*, *m*) we generate from the COV-OBS.x2 spline model coefficients a vector $\mathbf{y} ^o$ that contains $g_n^m$ values at $N^o$ epochs $t^o_j$ spanning $[t_s^o,t_e^o]$ every $\Delta t^o$. Observation error variances ${\sigma _{g_n^m}^{o2}}(t^o_j)$ for each coefficient are extracted from the COV-OBS.x2 posterior covariance matrix, and stored into a diagonal matrix ${\textsf {R}_{\textsf {yy}}}$.

We wish to estimate a vector $\mathbf{x}$ that contains analyzed Gauss coefficients $g_n^{ma}$ at $N^a$ epochs $t^a_j$ spanning $[t_s^a,t_e^a]$ every $\Delta t^a=1$ years (so that $N^a=(t_e^a-t_s^a)/\Delta t^a+1$), together with its associated uncertainties. To this purpose, we construct cross-covariance matrices ${\textsf {C}_{\textsf {xy}}}$, ${\textsf {C}_{\textsf {xx}}}$ and ${\textsf {C}_{\textsf {yy}}}$, of sizes, respectively, $N^a\times N^o$, $N^a\times N^a$ and $N^o\times N^o$, whose elements are

16 $$\begin{aligned} \left\{ \begin{array}{rl} {\textsf {C}_{\textsf {xy}}}(i,j) = &{}\displaystyle E\left( \left( g_n^m(t^a_i)-{\bar{g}}_n^m\right) \left( g_n^m(t^o_j)-{\bar{g}}_n^m\right) \right) \\ {\textsf {C}_{\textsf {xx}}}(i,j) = &{}\displaystyle E\left( \left( g_n^m(t^a_i)-{\bar{g}}_n^m\right) \left( g_n^m(t^a_j)-{\bar{g}}_n^m\right) \right) \\ {\textsf {C}_{\textsf {yy}}}(i,j) = &{}\displaystyle E\left( \left( g_n^m(t^o_i)-{\bar{g}}_n^m\right) \left( g_n^m(t^o_j)-{\bar{g}}_n^m\right) \right) \end{array} \right. \,. \end{aligned}$$

In the above definitions, the background value, denoted by overlines, is non-zero for the axial dipole $g_1^0$ only.

The model $\mathbf{x}$ thus results from the BLUE as

17 $$\begin{aligned} \mathbf{x} = {\bar{\mathbf{x}}} + {\textsf {C}_{\textsf {xy}}}({\textsf {C}_{\textsf {yy}}}+{\textsf {R}_{\textsf {yy}}})^{-1}(\mathbf{y} ^o - {\bar{\mathbf {y}}}) = {\bar{\mathbf {x}}} + {\textsf {K}_{\textsf {xy}}}(\mathbf{y} ^o - {\bar{\mathbf {y}}})\,, \end{aligned}$$

where ${\textsf {K}_{\textsf {xy}}}$ is the Kalman gain matrix, and ${\bar{\mathbf {x}}}$ (resp. ${\bar{\mathbf {y}}}$) is a vector of size $N^a$ (resp. $N^o$) filled with the background value ${\bar{g}}_n^m$. Cross-covariances of the uncertainties on the analyzed vector $\mathbf{x}$ are then given by the posterior covariance matrix

18 $$\begin{aligned} {\textsf {R}_{\textsf {xx}}} = {\textsf {C}_{\textsf {xx}}} - {\textsf {K}_{\textsf {xy}}}{\textsf {C}_{\textsf {xy}}}^T\,. \end{aligned}$$

For details about the above estimation procedure (also known as kriging method, Optimal Interpolation, Gaussian interpolation, or Least-Squares Collocation) we refer for instance to Rasmussen and Williams (2006). To sample the dispersion of $\mathbf{x}$, an ensemble of *k* realizations is generated from the Cholesky decomposition of ${\textsf {R}_{\textsf {xx}}}$ (see Gillet et al. 2013). The ISTerre candidate models for IGRF-13, together with their associated uncertainties, have been derived based on the methodology described above

## Results and discussion

### The COV-OBS.x2 field model

#### Statistics on prediction errors

**Table 2:** Errors statistics for the GO and VO (CHAMP and Swarm) datasets integrated in COV-OBS.x2: accepted number of data $N_o$, fraction of rejected data ($N_{o}^{*}$, in %), dimensionless L2 data misfit ${\mathcal{M}}^*$ and bias $\mu ^*$, and dimensional L2 data misfit ${\mathcal{M}}$ and bias $\mu$

Dataset	$N_o$	$N_o^*$ (%)	${\mathcal{M}}^*$				$\mu ^*$				${\mathcal{M}}$				$\mu$			
			*X*	*Y*	*Z*	*F*	*X*	*Y*	*Z*	*F*	*X*	*Y*	*Z*	*F*	*X*	*Y*	*Z*	*F*
GO	21056	5.57	1.27	0.79	1.11	$-$	$-$0.01	0.00	$-$0.04	$-$	4.55	2.87	4.22	$-$	$-$0.01	$-$0.01	$-$0.15	$-$
CHAMP	17525	5.17	1.28	1.02	1.04	0.99	0.01	0.04	0.10	0.05	3.42	2.59	2.57	3.04	0.04	0.11	0.28	0.15
Swarm	13354	3.77	1.31	0.85	0.94	0.92	0.10	$-$0.04	0.11	0.08	3.14	2.11	2.10	2.69	0.28	$-$0.05	0.25	0.23
Total	51935	4.98	1.29	0.89	1.04	0.96	0.02	0.01	0.04	0.06	3.88	2.61	3.28	2.89	0.08	0.02	0.09	0.19

Dimensional misfits and biases are in units of nT for VOs, and nT/year for GO

**Fig. 3:**
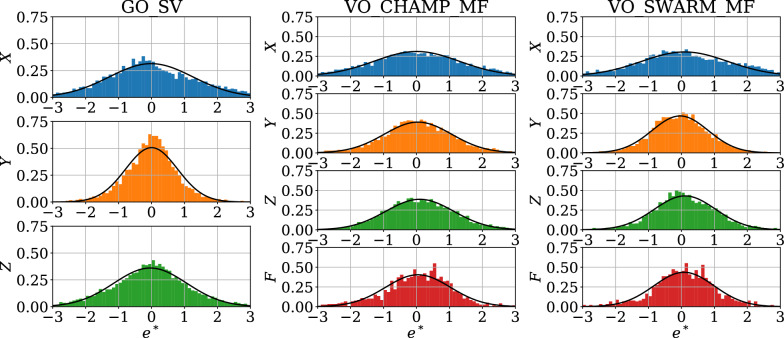
Histograms of the normalized data misfit $e^*$ for the GO and VO datasets (separated all three components). In black is the normalized Gaussian curve ${\mathcal{N}}(\mu , \sigma )$ with $\mu$ the mean and $\sigma$ the standard deviation of the normalized misfits (see Table 2)

**Fig. 4:**
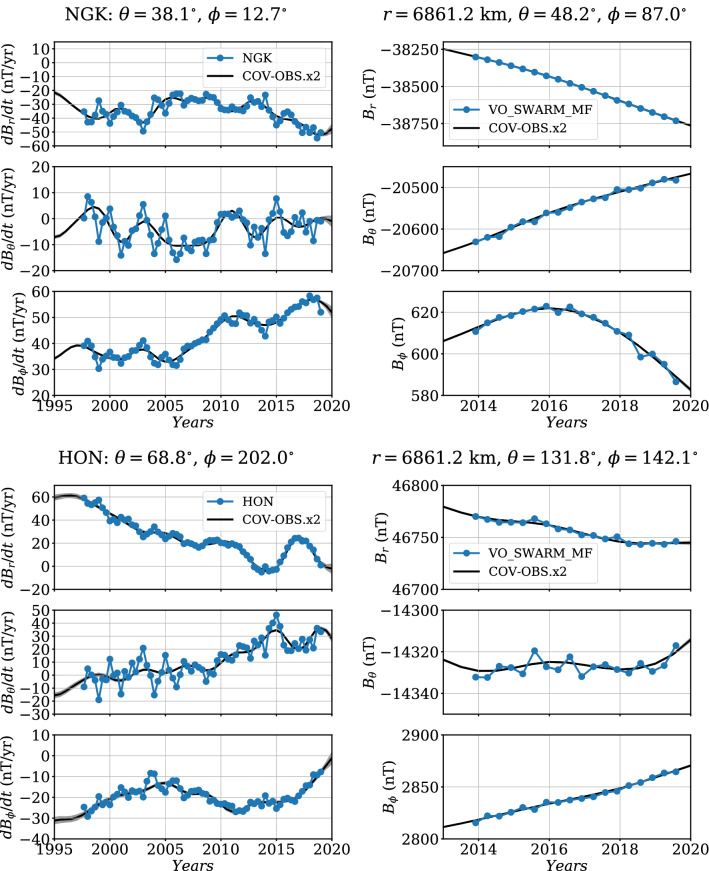
Predictions from COV-OBS.x2 for the three geocentric components. Left: SV at the Niemegk (NGK, top) and Honolulu (HON, bottom) observatories. Right: MF at two examples of Swarm VO in the Northern (top) and Southern (bottom) hemispheres

We provide in Table 2 some statistics concerning the COV-OBS.x2 misfits and biases to the new (GO and VO) data sets, separately for all three components. Our algorithm rejects only a small part of the dataset ($\approx 5\%$ in average). We consider the normalized L2 data misfit and bias,

19 $$\begin{aligned} {{{\mathcal{M}}}}^{*}=\sqrt{\frac{1}{N_o}\sum _k {e_k^*}^2} \;\;\mathrm {and}\;\; \displaystyle \mu ^*=\frac{1}{N_o}\sum _k e_k^*\,, \end{aligned}$$

for $e_k^*$ the normalized prediction error for the *k*th datum (we also consider the dimensional L2 data misfit ${{{\mathcal{M}}}}$ and bias $\mu$). For all three datasets and all components, no significant bias is found, as all normalized biases $\mu ^*$ remain close to zero. Normalized L2 misfits are reasonably close to unity (in average slightly weaker on *Y* for GO and Swarm data, and bit larger on *X* for all three data sources). Dimensional misfits, typically a few nT (or nT/year) on all three components, are a bit larger on *X* (and on *Z* for GO). Dimensional averaged biases are for all components of all datasets less than $\approx 0.3$ nT. We present in Fig. 3 the distribution of the normalized residuals (VO and GO) for all three components. These are reasonably close to Gaussian, although sometimes slightly more peaked (see for instance on *Y* for the GO SV data). We also notice some slight asymmetry in the shape of some residuals distributions (e.g., the *X* component on Swarm and GO data). In this context where normalized misfits (resp. biases) are close to 1 (resp. 0) and where the PDF of normalized residuals is close to a ${{{\mathcal{N}}}}(0,1)$ Gaussian distribution, we consider that the obtained posterior model uncertainties (based upon the inverse Hessian matrix, see Gillet et al. 2013) constitute reasonable errors estimates. In order to further illustrate the fit to GO and VO series, we give in Fig. 4 two examples of our average model SV predictions on ground, and MF predictions at Swarm’s altitude.

#### COV-OBS.x2 over the satellite era

**Fig. 5:**
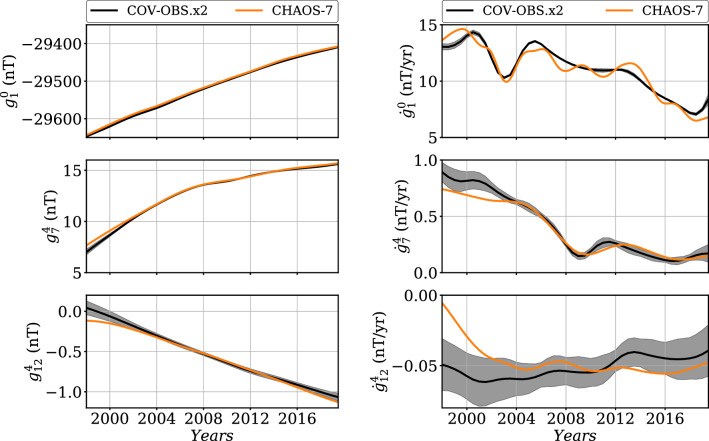
MF (left) and SV (right) time series of $g_1^0$ (top), $g_7^4$ (middle) and $g_{12}^{4}$ (bottom) for COV-OBS.x2 (black), compared with CHAOS-7 (orange) between 1998 and 2020. The gray-shaded areas represent the $\pm \sigma$ dispersion within the ensemble of COV-OBS.x2 models

We illustrate in Fig. 5 the time evolution of MF and SV Gauss coefficients for COV-OBS.x2 over the era covered by VOs. As observed by Gillet et al. (2015) with COV-OBS.x1, their evolutions are overall coherent with that of the CHAOS-7 model (Finlay et al. 2020), put aside an $\approx 2$ nT shift on $g_1^0$ in link with the differences of induced model. However, we notice that

i. We do not capture some of the rapid changes (of period less than $\approx 2$ years) for the larger length-scales, due to the use of 2 years knot spacing for the splines basis;
ii. Consequence of the employed stochastic prior, our solutions tend to be less smooth towards small length-scales.

The COV-OBS.x2 uncertainty estimates most often encompass the difference with CHAOS-7 towards small length-scales (except towards the beginning of the time-span covered by CHAOS-7).

#### Unmodeled rapid field changes

We have seen that the use of a cubic B-splines basis with knots separated by 2 years does not permit the capture of short time-scales features. As a consequence, COV-OBS.x2 uncertainties only represent errors on Gauss coefficients low-pass filtered (at periods longer than $\approx 2$ years). If used for comparison with instantaneous pictures of the core dynamics, these should be complemented by an error estimate that accounts for unmodeled rapid field changes. This latter will supplement COV-OBS.x2 formal errors, especially at the largest length-scales.

**Fig. 6:**
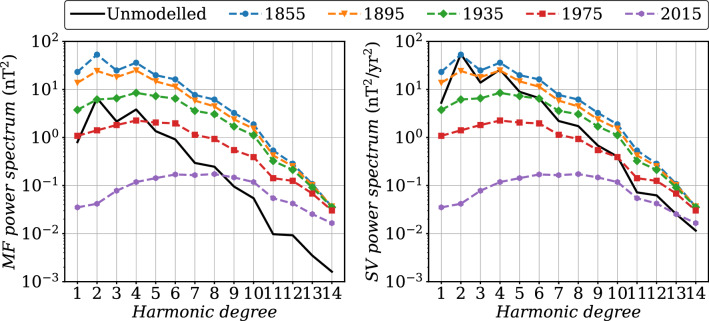
In black: MF (left) and SV (right) Lowes spectra (Eq. (3)) of our estimate of the unmodeled internal field at high frequencies due to the projection onto splines 2 years apart. It is constructed from the residuals to a spline fit to synthetic AR-2 series (see text for details). In color (dotted lines) are shown the spectra for the COV-OBS.x2 formal posterior uncertainty at epochs 1855, 1895, 1935, 1975, and 2015. These latter are obtained from the $\pm \sigma$ spread within an ensemble of COV-OBS.x2 realizations (i.e., projected onto splines)

To illustrate this issue, we estimate the magnitude of signals unable to be represented by the B-splines basis. To do so, we generate a set of synthetic Gauss coefficient series with spectral properties defined by the AR-2 stochastic prior considered in this study. We then fit cubic B-splines, with knots 2 years apart, to each of these coefficient series, and consider the residuals between the original synthetics and the fitted series as the unmodeled high frequency signal. We show in Fig. 6 the time average MF and SV Lowes spectra for these residuals, compared with COV-OBS.x2 formal uncertainties derived from the spline coefficients posterior covariance matrix. The contribution of unmodeled rapid field changes appears negligible towards high harmonic degrees. At large length-scales however, its power is larger than that of the COV-OBS.x2 formal errors, in particular during the satellite era. It is for instance of the order of 1 nT/year for SV dipole coefficients, comparable to the differences observed on $\partial _tg_1^0$ between CHAOS-7 and COV-OBS.x2 in Fig. 5.

### Extracting IGRF candidate models from COV-OBS.x2 and its uncertainties

#### Validation of 5-year forecast from the BLUE

**Fig. 7:**
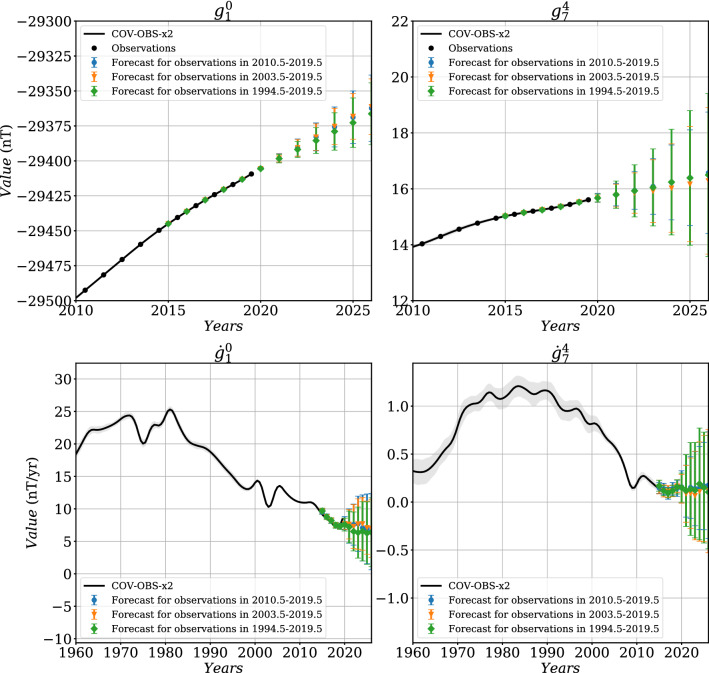
5-years forecasts using and $\Delta t^o=1$ year, for different observation periods, compared with COV-OBS.x2 in black (with in gray-shaded area the associated $\pm \sigma$ uncertainties), for the MF (top) and the associated SV (bottom) of Gauss coefficients $g_1^0$ (left) and $g_7^4$ (right). Errorbars represent the dispersion ($\pm \sigma$) with the ensemble of forecasts

**Fig. 8:**
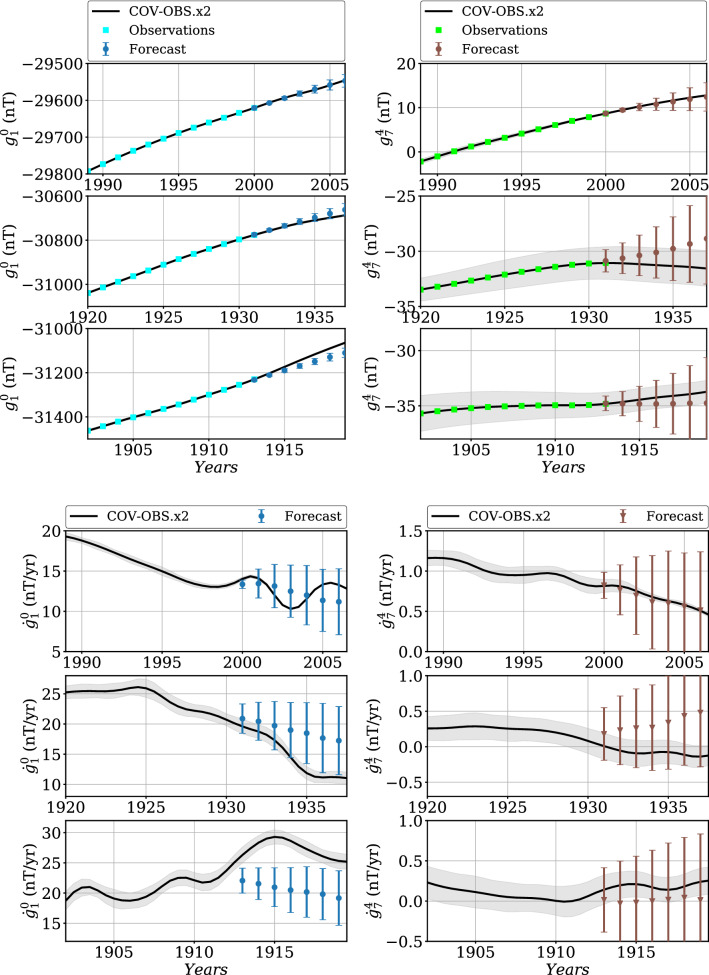
5-years forecasts using $\Delta t^o=1$ year, for different observation periods, compared with COV-OBS.x2 in black (and in shaded gray its $\pm \sigma$ dispersion), for the MF (top panels) and the associated SV (bottom panels) of Gauss coefficients $g_1^0$ (left) and $g_7^4$ (right). Errorbars represent the dispersion ($\pm \sigma$) within the ensemble of forecasts

We now apply our stochastic approach (the BLUE, see Methods) to the generation of 5-year predictions. We first test the effect of the observation period $[t_s^o,t_e^o]$ on the Gauss coefficient forecast over $[t_s^a,t_e^a]=[2015, 2025]$, by varying $t_s^o-t_e^o$ from 6 to 75 years, with $\Delta t^o=1$ years and $t_e^o=2019.5$. The SV forecast is obtained by first differentiating MF prediction series. We give in Fig. 7 the MF and SV forecasts of $g_1^0$ and $g_7^4$, together with their associated dispersion. Our investigations show that the length of the observation period has a relative little impact on the resulting forecast and its associated spread. This is certainly due to the nature of the employed AR-2 stochastic processes: discrete AR-2 processes have memory over only two successive dates: the correlation functions that enter matrices ${\textsf {C}_{\textsf {xy}}}$ and ${\textsf {C}_{\textsf {yy}}}$ play a major role on the dispersion within the ensemble of MF forecasts, which then evolves $\propto (t-t^o_e)^2$.

To assess the ability of the forecast spread to encapsulate the ‘true’ model trajectory, we test it over ancient periods covered by COV-OBS.x2 datasets, and perform 5-year forecasts. Drawing upon the above conclusion, $N^o=17$ observation epochs are used, sampled every $\Delta t^o= 1$ year. We show in Fig. 8 the obtained MF and SV predictions for Gauss coefficients $g_1^0$ and $g_7^4$, and for three observation periods ending at $t_e^o=t_s^a=$ 2000, 1931 and 1913, periods characterized by different behaviors in particular of the axial dipole (in all three cases $t_e^a=t_s^a+5$ year). Again, the SV forecast is obtained by first differentiating MF prediction series. For all Gauss coefficients but the axial dipole, MF and SV COV-OBS.x2 average model stay within $\pm \sigma$ of the forecast spread whatever the observation period chosen. For $g_1^0$ the forecast spread must sometimes be extended to about $\pm 2\sigma$, especially at epochs showing intense and monotonous trends in the dipole SV (see also Additional file 1). We overall consider that the stochastic forecasts are consistent with the COV-OBS.x2 past evolution, which validates the prediction using the BLUE method.

#### Application to field model predictions over 2015–2020

**Fig. 9:**
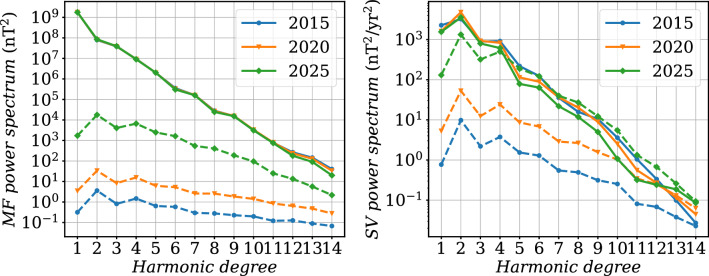
MF (left) and SV (right) Lowes spectra (Eq. (3)) at the Earth’s surface for epochs 2015, 2020 and 2025, estimated with the spline-free BLUE (see Methods). In dashed lines the respective spectra for the $\pm \sigma$ spread within the ensemble of models

We now apply the spline-free BLUE (see Methods) for the production of IGRF-13 candidate models, using $[t_s^a,t_e^a]=[2015, 2025]$. We present in Fig. 9 the MF and SV Lowes spectra obtained at the three epochs 2015, 2020, and 2025 of interest for the IGRF and DGRF model candidates. The MF dispersion spectrum is weaker in 2015 than in 2020 (at this latter epoch, data constraints are only from past epochs). It significantly increases for the prediction after 5 years without observations, to reach values only slightly above those documented for the IGRF-12 candidate model based on COV-OBS.x1 (Gillet et al. 2015). The MF spectrum is noticeably less in 2025 for the highest degrees: after the last available observation, the AR-2 stochastic prior brings the ensemble average MF estimate back to the background, in a time-scale faster for shorter wave-lengths – as expected given the shorter cut-off frequencies $\omega _n$ for large harmonic degrees *n*, see Eq. (8) and Gillet et al. (2013).

The SV spectrum for the ensemble average forecast decreases over time when no data is available. This reflects the fact that on average the stochastic prior drives the model back to the prior expectation (the ensemble average SV coefficients decay exponentially, over time-scales governed by the stochastic process parameters). The spectrum of the SV spread gradually increases over time from the last observation date $t_e^o$ (it behaves on short period as that of a random walk, i.e., $\propto \sqrt{t-t_e^o}$). Our present estimate of SV uncertainties is significantly larger than that documented in Gillet et al. (2015), in particular during the period with observations. This is primarily related with the spline-free estimate in the present study, which avoids under-estimating the effect of high-frequency SV changes (see also above the discussion of Fig. 6). Indeed we have checked that the inflation of data errors (see ‘Geomagnetic data’ section) only has a minor impact on the posterior model uncertainties. After 5 years without observations, the magnitude of spread within the ensemble of models is similar to that of the average model for degrees $n\ge 4$, illustrating the inability of the stochastic model (by construction) to deterministically predict the magnetic field evolution.

### Long-period variations in the external dipole field

**Fig. 10:**
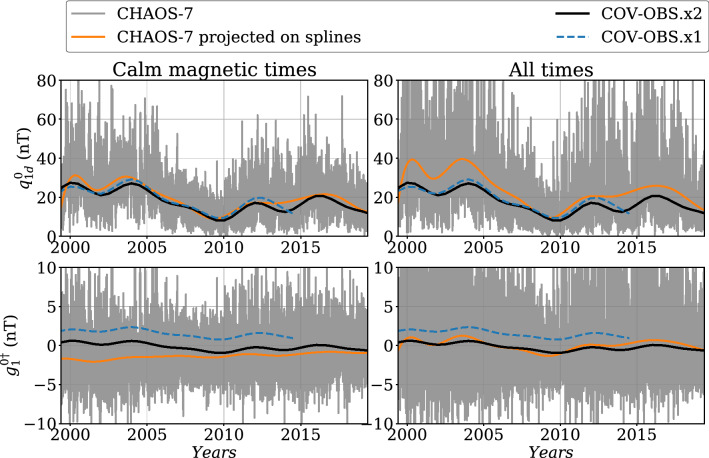
Time evolution of the external dipole field coefficient in dipole coordinates (top: $q_{1d}^0$) and induced dipole field coefficient in geocentric coordinates (bottom: $g_{1}^{0\dag }$) for COV-OBS.x2 (black), compared with the previous edition COV-OBS.x1 (dashed blue), superimposed with the corresponding estimate from CHAOS-7 (gray, selected over calm magnetic times on the left column (see text for details) and for all times in the right column) and its projection onto splines with knots 2-year apart (orange)

We now analyze the time evolution of the co-estimated parameter $q_{1d}^0$, the external axial dipole coefficient in internal dipole coordinates. As shown in Fig. 10 (top left), it agrees well with the CHAOS-7 estimate over the past two decades. We consider here low-pass filtered CHAOS-7 series, selecting only quiet geomagnetic times based on $K_p\le 30$ and $|dRC/dt|\le 2.1$ nT/hr. As such, if our model for $q_{1d}^0(t)$ under-estimates slow changes in $q_{1d}^0$ when averaged over all magnetic conditions (Fig. 10, top right), it is representative of the calm magnetosphere at periods longer than $\approx 2$ years. It is also very similar to the previous estimates from the COV-OBS.x1 model, despite a different processing of satellite observations (VO versus pointwise data in previous editions).

The associated induced field $g_{1d}^{0\dag }$ in geocentric coordinates presents a long period off-set in comparison with the corresponding coefficient for CHAOS-7, low-pass filtered and selected under quiet magnetic conditions (Fig. 10, bottom left). This is because an aliasing effect comes with the selection of calm periods. Indeed, the induced perturbation in CHAOS-7 presents by construction a zero-mean once averaged over all times, as seen in Fig. 10 (bottom right). This is also the case in COV-OBS.x2: as a consequence, $g_{1d}^{0\dag }$ is closer to the long-period induced field over all magnetic conditions (though with smaller fluctuations than in CHAOS-7). Contrary to what is done within the COV-OBS.x2 set-up (see section Methods), the background external field ${\overline{q}}_{1d}^0$ was accounted for when calculating the $g_{1}^{m\dag }$ in COV-OBS.x1 (see Eq. (15)). This results in the shift observed for $g_{1}^{0\dag }$ between these two models. All in all, the set-up for the induced field used for constructing COV-OBS.x2, even imperfect, reduces the shift to models dedicated to satellite observations such as CHAOS-7.

**Fig. 11:**
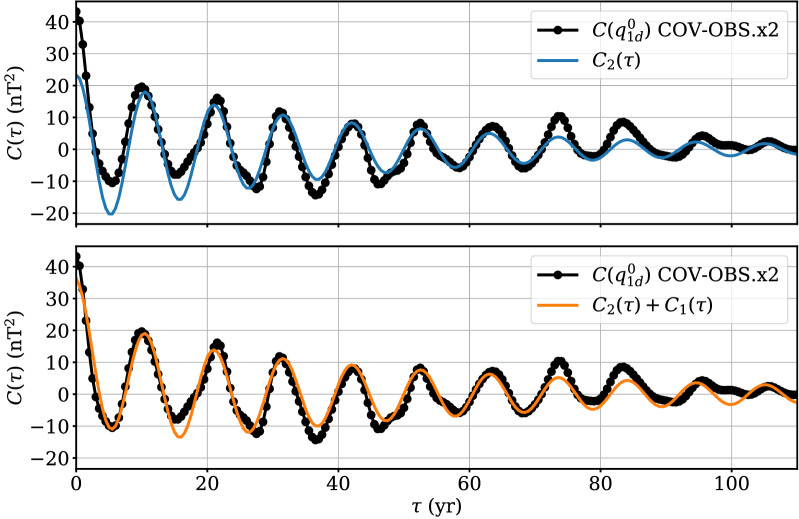
Auto-correlation function for the external dipole field coefficient $q_{1d}^0$ in dipole coordinates, superimposed with the fit obtained with (top) the 3-parameter function $C_2(\tau )$, and (bottom) a 5-parameter function $C_2(\tau )+C_1(\tau )$

**Table 3:** Parameters of the correlation functions $C_2(\tau )$ and $C_2(\tau )+C_1(\tau )$ fitted to $C_{q_{1d}^0}(\tau )$

Parameter	$\sigma _2^2$ (nT$^2$)	$1/\alpha _2$ (year)	$2\pi /\beta _2$ (year)	$\sigma _1^2$ (nT$^2$)	$1/\alpha _1$ (year)	$\chi _C$ (nT$^2$)
$C_2$	$23.3 \pm 1.2$	$41 \pm 4$	$10.51 \pm 0.03$	–	–	4.93
$C_2 + C_1$	$19.6 \pm 0.8$	$56 \pm 5$	$10.51 \pm 0.02$	$16.7 \pm 1.3$	$5.9 \pm 0.7$	3.08

We present in Fig. 11 the auto-correlation function $C_{q_{1d}^0}(\tau ) = E\left( {\tilde{q}}_{1d}^0(t){\tilde{q}}_{1d}^0(t+\tau )\right)$. It shows obvious oscillations of period $\approx 11$ years, in relation with the solar cycle. We fit (with the SciPy function *curve_fit* that uses a Levenberg–Marquardt method) $C_{q_{1d}^0}(\tau )$ with the three-parameter correlation function of a damped oscillator AR-2 process (Yaglom 1962):

20 $$\begin{aligned} C_{2}(\tau ) = \sigma _{2}^2\exp \left( -\alpha _{2}|\tau |\right) \left( \cos (\beta _2\tau )+\frac{\alpha _2}{\beta _2}\sin (\beta _2|\tau |)\right) \,. \end{aligned}$$

Within this formalism, $2\pi /\beta _2$ is the period of the oscillator, while $1/\alpha _{2}$ corresponds to a damping time. We use as ‘data’ annual values of $C_{q_{1d}^0}(\tau )$ over the period 1910–2020 (equivalent to $\approx 10$ solar cycles). These are weighted as $w(\tau )=\gamma (T-\tau )/T$, in order to down-weight the ill-constrained auto-correlations at long lags $\tau$, with $T=110$ year the maximum considered lag (a rather close fit is obtained using equal weights). We estimate $w(0)^{-1}=\gamma ^{-1}=E\left( \sigma ^2_{q^0_{1d}} -E\left( \sigma ^2_{q^0_{1d}}\right) \right)$ from an ensemble of the COV-OBS.x2 realizations, in order to have the uncertainty on the ‘data’ $C_{q^0_{1d}}(0)$ equal to the dispersion within the realizations of $\sigma ^2_{q^0_{1d}} = E\left( {\tilde{q}}^0_{1d}(t)^2\right)$.

The fit by $C_2$ recovers well an oscillation of period $2\pi /\beta _2\simeq 10.5$ years, with a decay rate $1/\alpha _2\approx 40$ years. However, it does not manage to capture the correlation observed at short lags (see Fig. 11, top for $\tau < 10$ year). In particular, it underfits by a factor of about 2 the variance $\sigma ^2_{q_{1d}^0} = C_{q_{1d}^0}(0)$ (see Fig. 11). In order to reduce this inconsistency, we now consider on top of the damped oscillator (described by $C_2$) an independent auto-regressive process of order 1 (a damped random walk), whose two-parameter correlation function $C_1$ is

21 $$\begin{aligned} C_{1}(\tau ) = \sigma _{1}^2\exp \left( -\alpha _{1}|\tau |\right), \end{aligned}$$

and now fit $C_{q_{1d}^0}(\tau )$ with $C_{1}(\tau )+C_{2}(\tau )$. $1/\alpha _{1}$ corresponds here to the characteristic memory time of the random walk process. The addition of this independent process reduces the inconsistency at short lags (see Fig. 11, bottom), by adding a Laplace correlation with decay time $1/\alpha _1\approx 6$ year. More quantitatively, it significantly reduces the misfit as measured by

22 $$\begin{aligned} \chi _C^2 = \frac{\displaystyle \int _{\tau =0}^T w(\tau )(C_{q_{1d}^0}(\tau )-C^*(\tau ))^2 d\tau }{\displaystyle \int _{\tau =0}^T w(\tau ) d\tau }\,, \end{aligned}$$

and reported in Table 3 for $C^*=C_2$ or $C_2+C_1$. The fitted variance of $q_{1d}^0$ is approximately evenly shared between the above AR-1 and AR-2 processes (it is, respectively, $\approx 17$ and 20 nT$^2$). The damped oscillator period, $2\pi /\beta _2\simeq 10.5$ year, is not affected by the addition of the AR-1 process. However, its decay time $1/\alpha _2\approx 55$ years is significantly larger than the value obtained with the AR-2 correlation function alone (see Table 3). In this latter case, $1/\alpha _2$ was likely biased towards short value, because the damped oscillator model alone is designed to accommodate both the large covariance at short lags (but in practice fails) and the much smaller values at long lags. This inconsistency is relaxed when adding on top an AR-1 process, leading to a larger estimate of the damped oscillator decay time.

Such multi-decadal decorrelation may be attributed to the natural cycle to cycle variability in duration and amplitude (see the reviews by Petrovay 2010; Hathaway 2015), also possibly involving longer period modulations (Usoskin et al. 2007). Interestingly, while double maxima appear in some solar cycle indices (as for instance the sunspot number, see Petrovay 2010, Fig. 8), and while higher frequency oscillations show up in our $q_{1d}^0$ series (see Fig. 10), we do not recover any harmonic of the 10.5 years cycle in the correlation function (and fitting $C_{q_{1d}^0}$ with two AR-2 parameters performs less well than with the above function $C_2+C_1$).

## Conclusions

We produce the COV-OBS.x2 geomagnetic field model, which extends to 2020 previous generations of COV-OBS series of models. The primary data constraints used over recent epochs are annual differences of ground-based observatories’ series, and virtual observatories series from the CHAMP and Swarm satellite missions. The COV-OBS models not only propose the time evolution of Gauss coefficients, but as well an estimate of their uncertainties, based on temporal cross-covariances associated with stochastic processes.

We show how the COV-OBS approach can be used to propose a PDF for predictions of the MF and its SV, and illustrate it with 5-year forecasts, in the context of the IGRF-13 model. Over past epochs, the $\pm \sigma$ spread over 5 years encompasses the evolution of Gauss coefficients, except for the axial dipole. This coefficient is associated with a specific stochastic prior, characterized in the spectral domain by a range of frequencies where the temporal PSD of $g_1^0$ evolves as $f^{-2}$ (based on statistics from paleomagnetic records and observatory series). We consider in this study parameters that conservatively reduce this range, and thus limit the power at short periods (see Additional file 1). In currently available simulations, even in those proposed by Aubert et al. (2017), this range is further reduced (Aubert 2018; Gillet et al. 2019). For this reason, a one-to-one comparison of interannual changes in computations and geophysical observations cannot yet be performed, in link with Alfvén numbers relatively larger in geodynamo simulations.

Our model generally agrees well, over the satellite era, with regularized models such as CHAOS-7. The dispersion within our ensemble of models most often encompasses the difference between this model and the ensemble average COV-OBS-x2, at least at periods longer than two years. The use in COV-OBS models of a basis of cubic B-splines with knots separated by 2 years indeed does not allow shorter periods features to be captured. At first sight, not accounting for the above rapid changes in formal MF errors does not look critical. It may nevertheless facilitate conditions for instabilities when considering MF Gauss coefficients in data assimilation algorithms, such as the ones based on geodynamo equations by Sanchez et al. (2019). Considering explicitly the effect of unmodeled errors is also potentially important when using as data SV Gauss coefficients, as done for instance by Aubert (2014) with decorrelated snapshot estimates, or by Bärenzung et al. (2018) or Gillet et al. (2019) with reduced stochastic core flow models. This modification will be implemented in the pygeodyn assimilation tool by Huder et al. (2019). The fact that unmodeled errors are relatively larger for the SV than for the MF is due to the difference in their respective temporal spectra that shows a $-2$ slope for the SV and a $-4$ slope for the MF.

We then propose, as the COV-OBS.x2 instantaneous error estimate, the sum of (i) the formal error from spline coefficients (the one provided in previous versions of the COV-OBS model) and (ii) the uncertainty associated with the above estimate of unmodeled high-frequency signals. In the current work, we estimate the 5-year SV predictions from MF realizations obtained from a BLUE based on spline-free stochastic cross-covariances. We alleviate this way the under-estimation of SV uncertainties (see Fig. 7). This constitutes an improvement in comparison with Gillet et al. (2015), who use the formal posterior covariance matrix on spline coefficients.

## Supplementary information

**Additional file 1.** Impact of the 3 parameters for the axial dipole prior.

## Data Availability

Field models will be made available at the url https://geodyn.univ-grenoble-alpes.fr/.

## Abbreviations

MF: Main field
SV: Secular variation
IGRF: International Geomagnetic Reference Field
BLUE: Best linear unbiased estimate
PDF: Probability density function
VO: Virtual observatory
GO: Ground observatory
AR: Auto-regressive
GSM: Geocentric solar magnetospheric
SM: Solar magnetic
