# Supplementary material for: COV-OBS.x2: 180 years of geomagnetic field evolution from ground-based and satellite observations
Source: Earth Planets Space. 2020 Oct 23;72(1):160. doi: 10.1186/s40623-020-01194-2 (PMC7584567; doi:10.1186/s40623-020-01194-2)
Supplement: Supplementary file 1 — Additional file 1. Impact of the 3 parameters for the axial dipole prior. [file 40623_2020_1194_MOESM1_ESM.pdf]

Additional File

'COV-OBS.x2: 180 years of geomagnetic field evolution from ground-based and satellite observations',

https://doi.org/10.1186/s40623-020-01194-2

Earth, Panets & Space

by Loïc Huder[1], Nicolas Gillet[1†], Christopher C. Finlay[2], Magnus D. Hammer[2] and Hervé Tchoungui[1]

[1] Univ. Grenoble Alpes, Univ. Savoie Mont Blanc, CNRS, IRD, IFSTTAR, ISTerre, F-38000 Grenoble, France

[2] DTU Space, National Space Institute, Technical University of Denmark, Elektrovej 327, 2800 Kgs. Lyngby, Denmark

[†] Contact author: nicolas.gillet@univ-grenoble-alpes.fr

Appendix A. Impact of the 3 parameters for the axial dipole prior

The axial dipole prior is governed by three parameters: the variance σ and the two frequencies ω and χ, or alternatively σ and the time T_s and the SV variance $\sigma' = \sigma\omega$ (see the Methods section for details). In the context of building magnetic field models over the past 180 yrs, the crucial quantity is the power at decadal and shorter periods, which evolves as $T_s\omega^4\sigma^2/f^4 = T_s\sigma'^4/\sigma^2$. In this study, the cut-off period T_s is set to 100 kyr, as in Hellio & Gillet (2018), and the variance to $\sigma = 7700$ nT. Alternatively, Buffett et al. (2013) obtain, by fitting to PADM2M (Ziegler et al. 2011) or SINT2000 (Valet et al. 2005) linear stochastic models as described by Eq. (10), $T_s \approx 185$ kyr and values of σ ranging from 5300 to 7700 nT (depending on the considered model and time-span). For a given value of σ', decreasing σ increases ω. Consequently, we could have legitimately considered a prior authorizing a larger power on short periods dipole changes.

We illustrate this issue in Fig. 1, by comparing series of Gauss coefficients for different dipole prior values. The corresponding temporal PSD for the prior are shown in Fig. 2. When the data constraint is tight, all models coincide. However, SV axial dipole values differ towards the older epochs, and instabilities grow as the high frequency power is increased. The instability is particularly severe over the first 30 yrs of the model. There are nevertheless interannual oscillations, most likely unresolved, that potentially pollute the model further away from the starting point when a large power is assumed. This increase in

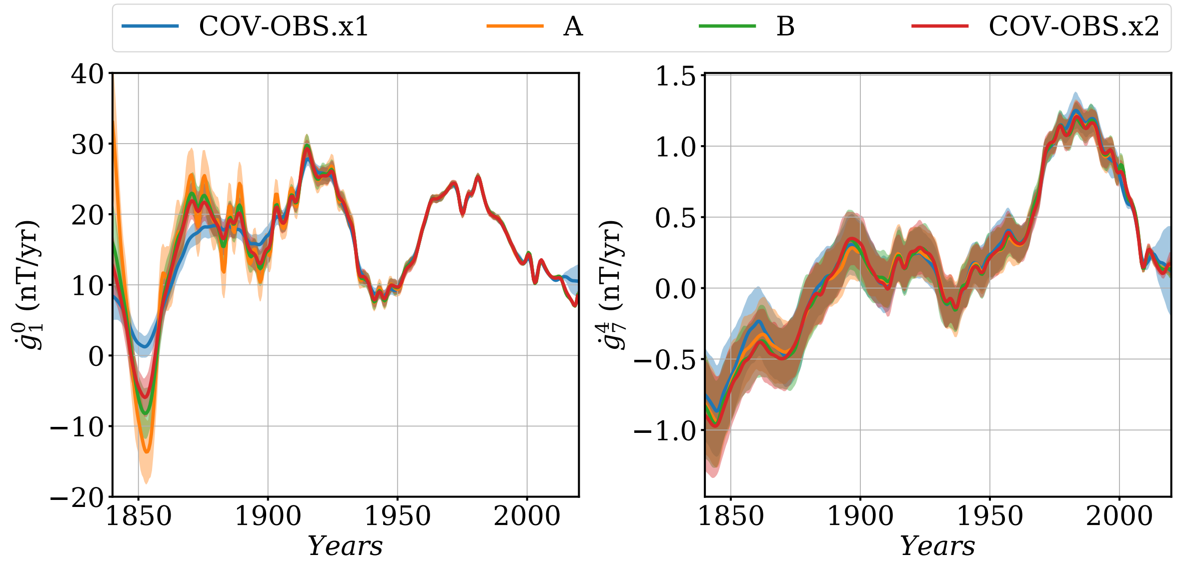

Figure 1. Time evolution of $\dot{g}_1^0$ (left) and $\dot{g}_7^4$ (right) for several AR-2 priors: case A ($\sigma = 6$ μT, $\omega^{-1} = 400$ yr, $\chi^{-1} = 20$ yr), case B ($\sigma = 6$ μT, $\omega^{-1} = 600$ yr, $\chi^{-1} = 45$ yr) and COV-OBS.x2 ($\sigma = 7.7$ μT, $\omega^{-1} = 770$ yr, $\chi^{-1} = 75$ yr). COV-OBS.x1 is also shown for comparison.

the model temporal complexity is indeed accompanied by no decrease in the normalized data misfit (see Fig. 3). Note that we observe no pollution of other Gauss coefficients associated with these oscillations. We motivated in the Method section our choice to go away from the 2-parameters prior for the axial dipole, and to distinguish it from other coefficients. However, we end-up here with a compromise in order to limit the observed instabilities. A way forward likely requires to abandon splines as support functions (as done by Hellio & Gillet 2018), as these possibly enhance the obtained oscillations. By considering nonlinear priors (e.g. Buffett & Puranam 2017), one will end-up with different parameter values, which may also render this issue less severe.

References

Buffett, B. & Puranam, A., 2017. Constructing stochastic models for dipole fluctuations from paleomagnetic observations, *Phys. Earth Planet. Int.*, **272**, 68–77.

Buffett, B. A., Ziegler, L., & Constable, C. G., 2013. A stochastic model for palaeomagnetic field variations, *Geophys. J. Int.*, **195**(1), 86–97.

Hellio, G. & Gillet, N., 2018. Time-correlation-based regression of the geomagnetic field from archeological and sediment records, *Geophys. J. Int.*, **214**(3), 1585–1607.

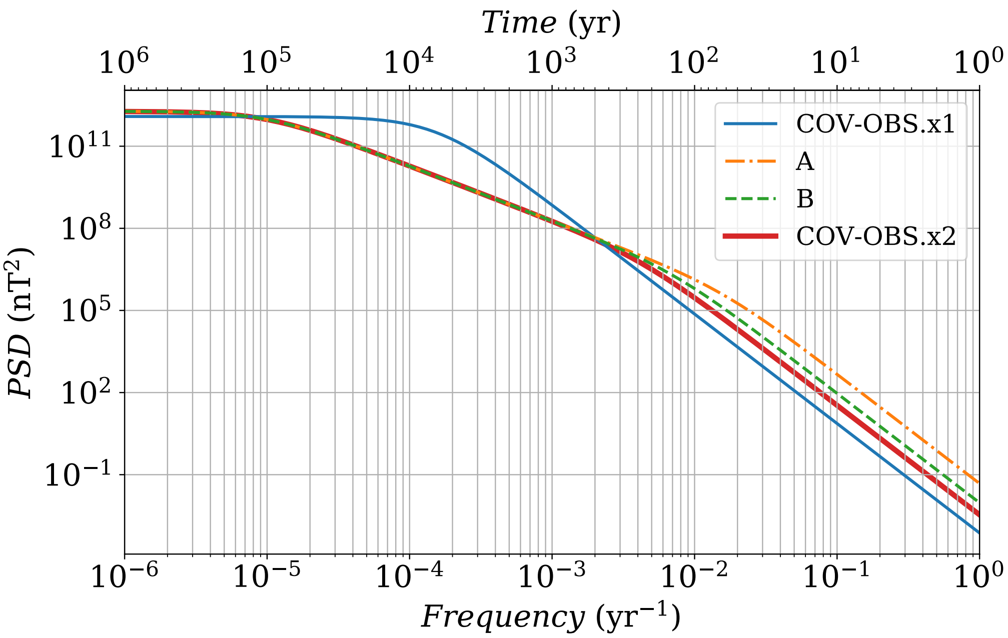

Figure 2. PSD spectra for several AR-2 priors: case A ($\sigma = 6$ μT, $\omega^{-1} = 400$ yr, $\chi^{-1} = 20$ yr, dash-dotted line), case B ($\sigma = 6$ μT, $\omega^{-1} = 600$ yr, $\chi^{-1} = 45$ yr, dashed line) and COV-OBS.x2 ($\sigma = 7.7$ μT, $\omega^{-1} = 770$ yr, $\chi^{-1} = 75$ yr, thick solid line). COV-OBS.x1 is also shown for comparison (thin solid line).

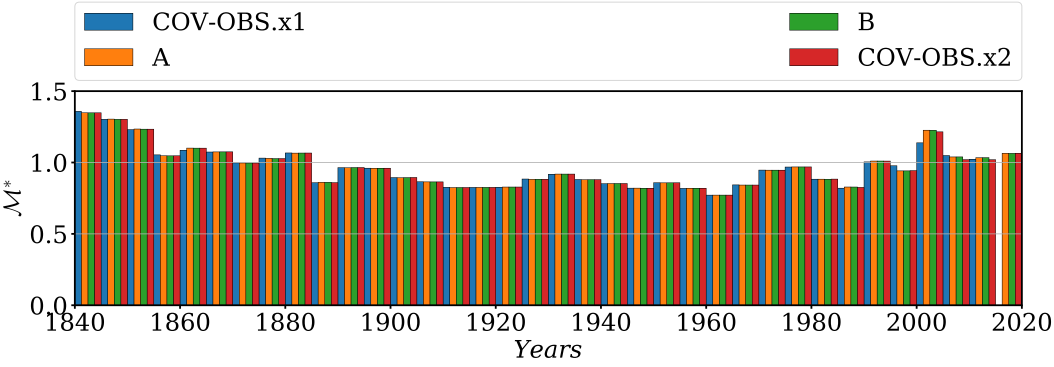

Figure 3. Normalized misfits and biases over 5-yr periods for several AR-2 priors: case A ($\sigma = 6$ μT, $\omega^{-1} = 400$ yr, $\chi^{-1} = 20$ yr), case B ($\sigma = 6$ μT, $\omega^{-1} = 600$ yr, $\chi^{-1} = 45$ yr) and COV-OBS.x2 ($\sigma = 7.7$ μT, $\omega^{-1} = 770$ yr, $\chi^{-1} = 75$ yr). COV-OBS.x1 is also shown for comparison.

Valet, J.-P., Meynadier, L., & Guyodo, Y., 2005. Geomagnetic dipole strength and reversal rate over the past two million years, *Nature*, **435**(7043), 802–805.

Ziegler, L. B., Constable, C. G., Johnson, C. L., & Tauxe, L., 2011. PADM2M: a penalized maximum likelihood model of the 0–2 Ma palaeomagnetic axial dipole moment, *Geophys. J. Int*, **184**(3), 1069–1089.
